# USE OF HIGH COST CARE AMONG VETERANS WITH COMORBID MENTAL ILLNESS AND ALZHEIMER’S DISEASE AND RELATED DEMENTIAS

**DOI:** 10.1093/geroni/igac059.1826

**Published:** 2022-12-20

**Authors:** Katherine Miller, Megan Shepherd-Banigan

**Affiliations:** University of Pennsylvania, Durham, Pennsylvania, United States; Durham VA Medical Center, Durham, North Carolina, United States

## Abstract

As Vietnam-era Veterans age, VA faces a growing need to manage aging-related conditions, such as Alzheimer’s disease and other dementia (ADRD), for this complex patient population. ADRD is of particular concern as military related factors, such as psychiatric illnesses, increase ADRD likelihood and complicate care management. In particular, decreasing use of low value care (e.g., emergency department (ED) care) is one approach to promote appropriate supportive care for Veterans with comorbid ADRD and psychiatric illness, but is understudied. We describe differences in potentially low value health care among older Veterans with ADRD 12 months after a new ADRD diagnosis. We compare Veterans with mental illness (MI) pre-ADRD diagnosis (major depressive disorder, post-traumatic stress disorder, or generalized anxiety disorder) to those without pre-existing MI. Potentially low value care includes ED use, hospitalization, and 30-day readmissions 12 months after a new ADRD diagnosis. Compared to Veterans with no pre-existing MI, Veterans with pre-existing MI are more likely to be younger (79 vs. 82 years old), male, married, White, Hispanic. In the year after new ADRD diagnosis, 16.9%, 21.6% and 2.6% of Veterans with MI had any ED visit, hospitalization, and 30-day readmission, respectively. In contrast, only 7.3%, 9.5%, and 0.9% of Veterans without pre-existing MI had any ED visit, hospitalization, and 30-day readmission, respectively. Our findings suggest that reducing low-value care may be an appropriate intervention target to improve care quality for Veterans with ADRD and mental illness.

